# Gauge-and-compass migration: inherited magnetic headings and signposts can adapt to changing geomagnetic landscapes

**DOI:** 10.1186/s40462-023-00406-0

**Published:** 2023-07-05

**Authors:** James D. McLaren, Heiko Schmaljohann, Bernd Blasius

**Affiliations:** 1 Institute for Chemistry and Biology of the Marine Environment (ICBM), University of Oldenburg, 26129 Oldenburg, Germany; 2 Institute for Biology and Environmental Sciences (IBU), Carl Von Ossietzky University of Oldenburg, 26129 Oldenburg, Germany; 3 Institute of Avian Research, 26386 Wilhelmshaven, Germany; 4 Helmholtz Institute for Functional Marine Biodiversity (HIFMB), University of Oldenburg, 26129 Oldenburg, Germany

**Keywords:** Animal migration, Evolutionary strategy algorithm, Migratory orientation program, Magnetic compass, Geomagnetic core field, Bet-hedging, *Zugknick*, Secular variation, Northern wheatear, Natal dispersal

## Abstract

**Background:**

For many migratory species, inexperienced (naïve) individuals reach remote non-breeding areas independently using one or more inherited compass headings and, potentially, magnetic signposts to gauge where to switch between compass headings. Inherited magnetic-based migration has not yet been assessed as a population-level process, particularly across strong geomagnetic gradients or where long-term geomagnetic shifts (hereafter, secular variation) could create mismatches with magnetic headings. Therefore, it remains unclear whether inherited magnetic headings and signposts could potentially adapt to secular variation under natural selection.

**Methods:**

To address these unknowns, we modelled migratory orientation programs using an evolutionary algorithm incorporating global geomagnetic data (1900–2023). Modelled population mixing incorporated both natal dispersal and trans-generational inheritance of magnetic headings and signposts, including intrinsic (stochastic) variability in inheritance. Using the model, we assessed robustness of trans-hemispheric migration of a migratory songbird whose Nearctic breeding grounds have undergone rapid secular variation (mean 34° clockwise drift in declination, 1900–2023), and which travels across strong geomagnetic gradients via Europe to Africa.

**Results:**

Model-evolved magnetic-signposted migration was overall successful throughout the 124-year period, with 60–90% mean successful arrival across a broad range in plausible precision in compass headings and gauging signposts. Signposted migration reduced trans-Atlantic flight distances and was up to twice as successful compared with non-signposted migration. Magnetic headings shifted plastically in response to the secular variation (mean 16°–17° among orientation programs), whereas signpost latitudes were more constrained (3°–5° mean shifts). This plasticity required intrinsic variability in inheritance (model-evolved σ ≈ 2.6° standard error), preventing clockwise secular drift from causing unsustainable open-ocean flights.

**Conclusions:**

Our study supports the potential long-term viability of inherited magnetic migratory headings and signposts, and illustrates more generally how inherited migratory orientation programs can both mediate and constrain evolution of routes, in response to global environmental change.

**Supplementary Information:**

The online version contains supplementary material available at 10.1186/s40462-023-00406-0.

## Introduction

Myriads of migrating animals undertake seasonal journeys across regional to cross-continental-scales [1]. For many migratory populations, seasonal routes are primarily mediated culturally, i.e., by collective and social cues [2, 3]. However, many long-distance migrants such as butterflies, sea turtles and night-migratory songbirds, migrate largely independently of others [4–6]. Based on experience, migrants can develop a map sense to navigate (reach known destinations from unfamiliar locations), for example by extrapolating magnetic field components ([7, 8] but see [9, 10]). Inexperienced (hereafter, naïve) independently-migrating animals are thought to rely strongly on endogenous migratory programs mediated by circannual/circadian timing and inherited compass headings [11–13]. In the simplest case, a “clock-and-compass” migrant with a single inherited heading would, depending on its primary migratory compass, follow a geographic (e.g., star), magnetic or gradually-shifting sun compass course [14–16], relative to the appropriate cue axis. With a magnetic compass heading, the travel direction relative to geographic North–South (N–-S) shifts per definition with any change in magnetic declination (the clockwise angle from true to magnetic N–-S). Note that for avian migrants, magnetic N and S are distinguishable by the vertical tilt (inclination) of the magnetic field, but this does not imply that direction varies with inclination [5, 11, 17]. Many migration routes are indeed potentially explainable by one or more compass courses [14, 15], contingent upon possessing sufficient compass precision [16] and ability to negotiate currents [18, 19]. However, many other migratory routes require distinct direction changes (often termed *Zugknicks* in bird migration), e.g., to avoid ecological barriers [20, 21], or to exploit favourable habitats [22, 23] or supportive current systems [8, 24].

The mechanisms underlying how naïve migrants reliably mediate critical direction changes along unfamiliar routes remain unclear. Purely clock-mediated direction changes could prove unreliable given inherent variability in migratory schedules [25, 26]. Alternatively, naïve migrants could potentially take advantage of the broad-scale latitudinal structure of the Earth’s magnetic field. From polar to equatorial latitudes, magnetic inclination (hereafter, inclination) decreases from about 90°–0° in the N Hemisphere (increasing from − 90° to 0° in the S Hemisphere), and the total field intensity (hereafter, intensity) decreases from approximately 65,000–30,000 nT [9, 27] in both Hemispheres. Experimental evidence suggests that naïve migratory sea turtles, salmonids [8] and birds [11, 28] indeed can use geomagnetic information to mediate orientation shifts, though is inconclusive regarding which magnetic components are used and whether switches in headings are either extrapolated in situ or predetermined (note that to determine flight direction for a “known” magnetic compass heading, birds require a discernible vertical tilt but not necessarily a precisely measurable inclination angle [5, 11, 17]). Naïve migrants could potentially travel between inherited magnetic “signature” locations by extrapolating gradients in bi-coordinate geomagnetic components, e.g., inclination and intensity [8]; however, temporal geomagnetic variability and near-collinear component gradients could render such a strategy unreliable [9, 10, 29]. Alternatively, naïve migrants could mediate switches (*Zugknicks*) between a fixed sequence of inherited compass headings upon passing (gauging) inherited magnetic signposts, e.g., a threshold value of either inclination or intensity [8, 11]. In this study, we assess such a “gauge-and-compass” migratory orientation program, which does not require migrants to reach a specific geomagnetic location, nor to extrapolate between experienced geomagnetic configurations.

**Fig. 1:**
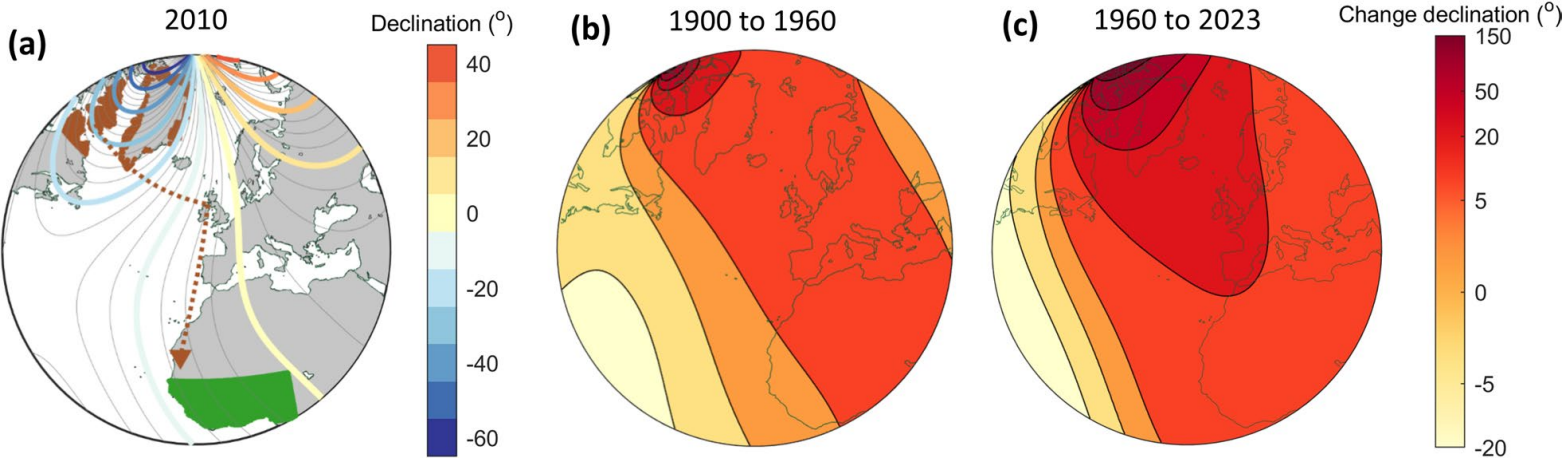
Extreme geomagnetic gradients and temporal shifts, illustrated for bird migration across the North Atlantic. **a** Contours of mean-field geomagnetic declination in October 2010 (degrees clockwise from true to magnetic North; colour scale on right), with modelled natal range (in brown) and wintering grounds (goal area; green) of northern wheatears (*Oenanthe oenanthe leucorhoa*). The brown dashed line and arrow depicts the approximate actual route from Iqaluit, Baffin Island taken by a juvenile (*leucorhoa*) northern wheatear tagged in 2010 using light-level geolocation [34]. Distance-minimizing great circle routes are straight lines in the stereographic projection [35]. **b**, **c** Contours of temporal change (secular variation) in mean-field declination from **b** 1900–1960 and **c** 1960–2023, with colour scale on right. Magnetic data are from a global IGRF modelled data of the Earth’s core-field [36, 37]

A critical factor affecting feasibility of magnetic compass-based movement is its robustness to spatiotemporal geomagnetic variability. The Earth’s magnetic field is irregularly aligned with the true geographic N–S axis, according to magnetic declination, and moreover undergoes temporal fluctuations between daily and centuries-long time scales [9, 27]. The overall pattern is largely attributable to the so-called core field, created by motion of magnetic fluid within the Earth’s inner core, with changes in the core field—termed secular variation—dominating geomagnetic variability at > 1-year scales [9, 30]. An example of extreme secular variation is found in East-arctic Canada and Greenland (Fig. 1a), where the N magnetic pole is drifting an order of magnitude faster than a century ago [31], with temporal shifts in declination sometimes exceeding 1° clockwise per year (Fig. 1b, c). Additionally, near-surface ferromagnetic material creates persistent magnetic anomalies to the core field, known as the crustal field; these are typically < 1000 nT, i.e., < 2% of mid-latitude field intensities [9]. Finally, solar and lunar activity cause within-daily to decadal variation including near-weekly magnetic storms; these typically last a few hours with peak intensity < 200 nT, but occasionally several weeks, and with peaks exceeding 1000 nT [9, 27]. Variability in declination and inclination in storms and crustal anomalies typically remains small (< 2°) [9, 32, 33].

It is often thought that compass-based movement through strongly varying geomagnetic fields is intractable without regular calibration using non-magnetic cues [9, 38, 39]. However, it is also underappreciated that broad spatial geomagnetic gradients could actually enhance the feasibility and efficiency of compass-based movement [35]. For example, with an Eastward increase in declination (as in Fig. 1), Southwest magnetic headings will partially correct for erroneous displacement (see Additional file 1: Fig. S1), e.g., by wind [40]. Nonetheless, in considering long-term robustness of inherited magnetic-based orientation, it is important to consider whether plasticity in inherited headings [12, 41, 42] can track secular variation at a population level. Since migratory headings vary geographically [16, 41, 43], and are often inherited as averages ([12, 41], but see [43]), it is additionally important to account for population-level mixing through natal dispersal (the distance from natal to subsequent breeding locations [44, 45]). More generally, intrinsic stochastic variability in inheritance of traits—as distinct from within-population variation—is expected to be beneficial in unpredictably varying environments [46–48]. That is, imperfect trait inheritance, sometimes referred to as bet-hedging, can actually enhance long-term population fitness despite reducing yearly expected mean fitness [42, 49].

To assess the long-term viability of inherited magnetic-based migration at the population level, including the benefit of inherited magnetic signposts, we developed a simulation model of migration through spatiotemporally-varying geomagnetic landscapes, using an evolutionary algorithm approach [50, 51]. The migration model is based on compass-based movement [16], extended to include geomagnetic-signposted directional switches in migratory direction. To focus on inheritance of migratory orientation rather than population demographics, we assessed individual fitness by only successful arrival in the modelled wintering grounds. We considered 124 years (1900–2023) of global modelled IGRF geomagnetic data [37] which closely approximates the time-mean field, at least over the last 40 years with available satellite magnetic data (standard errors in inclination, 0.3°, intensity, 180 nT, and declination, 0.4° [30]). The evolutionary algorithm mimics inheritance of migratory orientation among successful migrants, accounting for both spatial population mixing (through natal dispersal) and intrinsic variability in inheritance [42, 48]. We first performed a model spin-up, analogously to in climate models [18, 52], to create a viable test population for the migration route and time-period considered (hereafter, viable population), i.e., one adapted to both geomagnetic data from random years and the initial test year (1900). We then tested the robustness of the resultant viable population to geomagnetic change over the 124-year period. The model spin-up estimated optimal headings and signposts for 1900, optimal magnitudes of intrinsic variability (standard deviations) in inheritance of headings, signposts, and optimal (mean) natal dispersal. To assess the benefit of intrinsic variability in inheritance, as an uncertainty analysis, we additionally simulated migration with perfect inheritance (i.e., exact averaging of parental headings and signposts).

We considered three inherited migratory orientation programs (hereafter, orientation programs) for naïve bird migrants: non-signposted migration, i.e., following a single inherited heading, and two signposted migratory programs, based on either magnetic inclination or intensity. With an inherited signpost, modelled migrants shift to a second inherited (*Zugknick*) compass heading once the perceived (gauged) magnitude in inclination or intensity falls below an inherited threshold value. Using the viable population as a starting point, each migratory orientation program was assessed by arrival success over the 124-year simulated period. Our default precision in gauging intensity (2%) and inclination (5°) fell well within the typical ranges of both non-secular geomagnetic variability [9, 53], and estimated precision in magnetoreception [17, 54, 55]. As a sensitivity analysis, we separately assessed broader ranges in migrant precision, both in gauging magnetic field components and in overall flight-step direction [16]. Finally, as an uncertainty analysis, we tested declination-signposted migration, which would require extrapolation between a geomagnetic and geographic reference ([16], e.g., via a star or sun compass).

We chose to model a migratory songbird, the East-Nearctic-breeding population of the northern wheatear (*Oenanthe oenanthe leucorhoa*, hereafter *leucorhoa* wheatear). This subspecies faces a clear energetic and survival bottleneck, the Atlantic Ocean, *en route* to their wintering grounds in sub-Sahelian West Africa [20, 34], while also traversing strong geomagnetic gradients in a rapidly-drifting polar geomagnetic landscape (Fig. 1, [31]). Due to technological and practical limitations in tagging and tracking small birds in remote regions, the exact routes taken by *leucorhoa* wheatears remain uncertain [34, 56]. They potentially reach Spain or even Africa in several days of non-stop flight [40, 56], but are better known to detour (*Zugknick*) via North-West Europe, with a single tracked migration (Fig. 1a) from Baffin Island via Britain or Ireland [34], ringing recoveries from Southwest Greenland in France and Spain [56, 57], and multiple observations in the N Atlantic ocean [58, 59]. Following their trans-Atlantic flights, *leucorhoa* wheatears are night-migratory and open-habitat generalists, passing through Europe on a broad front [60]. To focus on orientation and geomagnetic effects, we implemented simple (and favourable) rules to locate nearby land when over water at dawn, and modelled flight energetics as flight capacity (potential flight hours). Flight capacity was replenished during extended stopover periods, i.e., times when birds rest, refuel and recover from endurance flights [61]. Stopovers occurred in any non-barren habitat, identified as Normalized Difference Vegetation Index, i.e., NDVI > 0 [62].

We by default modelled inherited magnetic headings (clockwise from magnetic N–S) which were re-determined on departures from stopovers (hereafter, as a primary compass) and maintained in-flight (hereafter, in-flight compass). For comparison, we tested alternative combinations of geographic and magnetic compass use, including where the primary compass is imprinted before migration or cue-transferred from a primary to an in-flight compass [16, 54]. With inheritance of geographic compass headings, a primary magnetic compass could still be imprinted, e.g., at the natal site [54]. With such an imprinted magnetic compass, offspring will "automatically" adjust for any between-year changes in secular variation (i.e., fly on average in the same geographic direction). Alternatively, with a primary celestial (star or sun) compass, an in-flight magnetic compass could be cue-transferred on departures from a primary geographic (star) or sun compass [16, 39]. Although such cue-transfers by naïve migrants likely compound orientation errors [16], for simplicity we assumed equivalent (default, 15°) compass precision when comparing combinations of compass use. Finally, we note that gauging of magnetic intensity or inclination signposts does not preclude a migrant from using a geographic (e.g., star) compass to determine flight headings.

As a predictive study, our models provide a strict test of both magnetic-based inheritance and signposted migration under geomagnetic change. Given the mortality risk over the ocean barrier, we predict that signposted migration benefits successful arrival of *leucoroha* wheatears in Africa. Furthermore, given the West–East gradient in declination (Fig. 1a, Additional file 1: Fig. S1), we expect that primary magnetic headings will be more successful compared with geographic (e.g., star compass) headings. Finally, we predict that inheritance of magnetic orientation benefits from intrinsic variability beyond natal dispersal and population mixing. More generally, our study highlights how natural selection might enable migratory populations to adapt to global changes in a key environmental migration cue.

## Methods

### Overview

We developed an evolutionary algorithm to model (micro-)evolution of inherited migratory headings and signposts, based on successful arrival of modelled naïve (first-fall) migrants to their wintering ground, and subsequent population mixing. Being specifically interested in assessing inherited migratory orientation (in maintaining arrival success) rather than population dynamics [63], we did not vary the population size or breeding (natal) locations, and considered only first-fall migration (i.e., spring migration is not modelled in our study). Therefore, every natal location was repopulated each year with a new (offspring) migrant based on two successful migrants from the previous year, selected randomly according to natal dispersal.

We assessed migratory orientation programs by long-term geometric mean in yearly arrival success, ${p}_{y}$, $\overline{p}={\left(\prod\nolimits_{y=1900}^{2023}{p}_{y}\right)}^{1/124}$. Geometric as opposed to arithmetic means are most appropriate for fitness or survival assessments, through accounting for disproportional negative effects of low success [42]. We also compared evolution of inherited headings, *Zugknick* signposts and locations, and kept track of mortality over water.

**Fig. 2:**
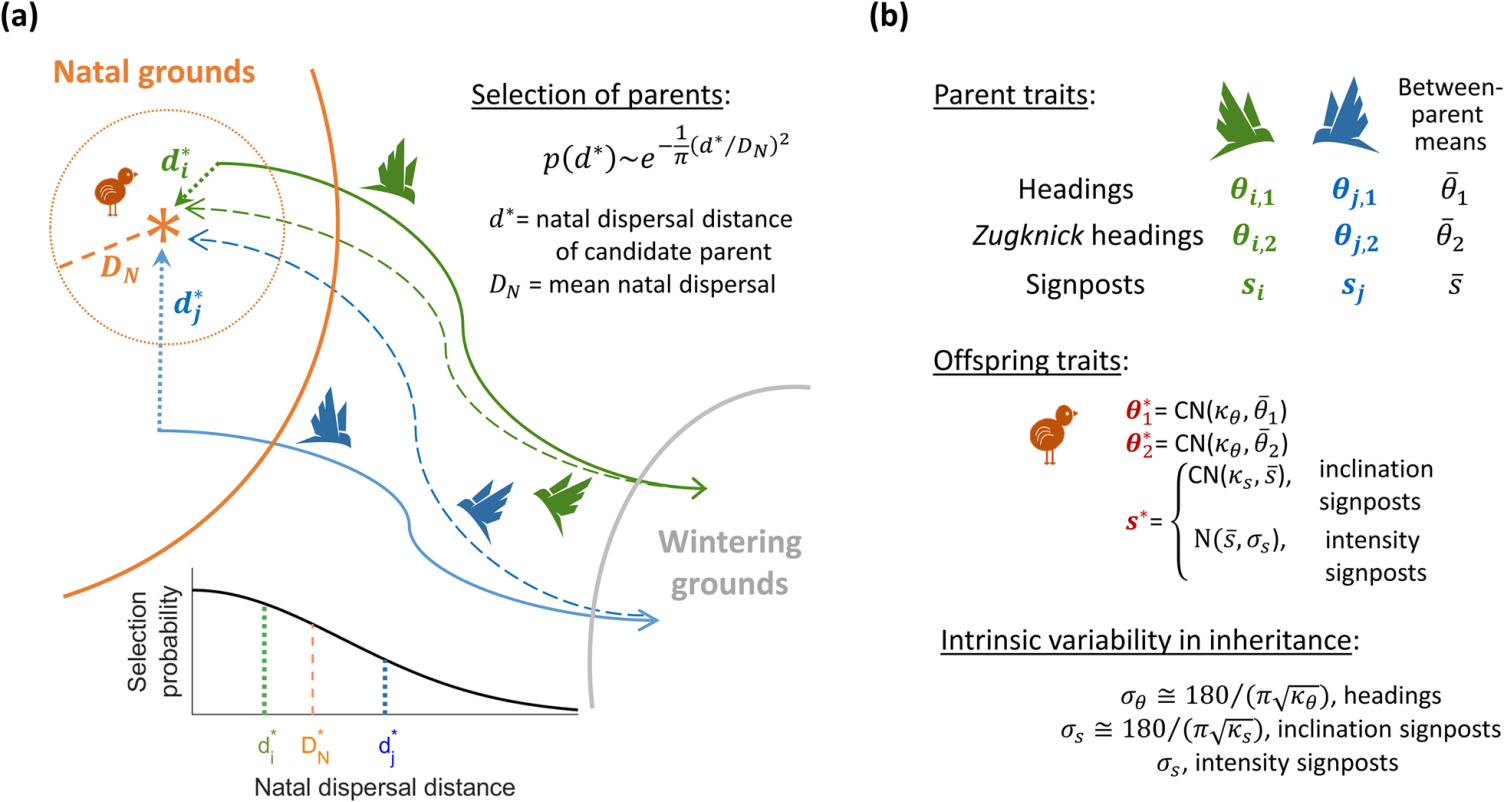
Modellled parent selection and trait inheritance (mixing) among successful migrants. **a** For each breeding location (orange asterisk) in each modelled year, two among all successful migrants were selected based on a sampled distance, $d^{*}$, between their natal and the focal breeding location relative to the population mean dispersal, *D*_*N*_ (illustrated as orange circle, with trajectories and sampled dispersal distances of two candidate “parents” depicted in blue and green). Selection probability followed a half-normal distribution (lower left graph, equation, top right). *D*_*N*_ was “evolved” as an individual trait during the initial model “spin-up” (see Additional file 2). **b** Next-generation (offspring) inheritance of migratory headings, $\theta_{1}^{*}$, $\theta_{2}^{*}$ and signposts, $s^{*}$, were sampled from circular von Mises distributions (CN, for headings and inclination signposts) and normal distributions (N, for intensity signposts), centred around between-parental means, together with intrinsic variability in inheritance for each trait (the latter also “evolved” during model spin-up; see Additional file 2). For non-signposted migration, only the first heading, $\theta_{1}^{*}$, is inherited

We first describe, in “Evolutionary algorithms” section, how we developed an evolutionary strategy algorithm to derive a viable migratory population and test its robustness to long-term geomagnetic change. Population mixing depended on two coupled processes: natal dispersal, and inheritance of headings and signposts, as described below and in Fig. 2. In “Migration model” section, we describe the simulated migration process, including determination of *leucorhoa* wheatear breeding range, flight headings, durations and energetics (fight capacity and replenishment at stopovers), and location of land when over water. In “Model implementation” section, we describe the geomagnetic and geographic data sources, software used and, in “Model consistency” section, sensitivity analyses to number of simulated individuals and stochastic replication.

Table 1 lists key model parameters and their attributes used both in the model spin-up (see below and [52]) and subsequent model simulations, including the default values, ranges or choices (e.g., in compass use) as well as sensitivity or uncertainty analyses. Angular quantities, i.e., headings and inclination (or declination) signposts, were sampled using a von Mises distribution, the circular equivalent of a normal distribution, governed by the von Mises concentration, κ [64, 65]. For interpretability, we report circular precision and variability by $\sigma =180/(\pi \sqrt{\kappa })$; this formula is nearly equivalent to circular standard deviation for σ < 30° [16, 65].

**Table 1:** Defaults, attributes and sampling of key model quantities for simulating inclination-signposted, intensity-signposted and non-signposted migration

Quantity	Default	Sampled	Distribution	Fixed or evolved	Sensitivity or uncertainty analysis
*Model simulations (following initial model spin-up, see below)*					
Population size	50,000	–	–	Fixed	1000–250,000
Duration	124 years (1900–2023)	–	–	–	Unchanging (1900) Backward (2023–1900)
Geomagnetic field	IGRF core-field [37]	Hourly (in-flight)	–	–	–
Natal locations	10°–80°W, 57.5°–84°N	Prior to 1st (spin-up) year	< 15% barren in 1° × 1° area, using Consensus Land Cover [62]	Fixed	–
Goal area (wintering grounds)	20°W–15°E, 5°–15°N	–	–	Evolved	–
Parent selection	Two	Yearly, at each location	Weighted by natal dispersal	–	–
Natal dispersal	Mean derived from spin-up (below)	Per location, among successful migrants	Half-normal (see Fig. 2)	Fixed (distribution)	Mean 10 m–250 km (also fixed in spin-up)
Inherited headings	Magnetic	Between selected parents	von Mises (see Fig. 2)	Evolved	Geographic (star) compass
Inherited signposts	Magnetic inclination, intensity	Between selected parents	von Mises or normal (see Fig. 2)	Evolved	Magnetic declination
Intrinsic variability in inheritance	σ or κ, from spin-up (below)	–	–	Fixed	No intrinsic variability (perfect trait averaging)
Primary compass	Magnetic	Per flight-step	von Mises	Fixed	Geographic (star) compass
In-flight headings	Magnetic	Hourly	von Mises	Fixed	Geographic (star) compass
Flight capacity	48–72 h	Replenished per stopover	Uniform	Fixed (range)	–
Flight-step precision	σ = 15° (κ = 14.6)	Per flight-step	von Mises	Fixed	5°–45° (κ = 1.6–131)
Precision gauging inclination	σ = 5° (κ = 131.3)	On landing	von Mises	Fixed	0.1°–20° (κ = 8–3.3 × 10^5^)
Precision gauging intensity	σ = 2%	On landing	Normal	Fixed	0.1–20%

Quantity	Range	Sampled	Distribution	Fixed or evolved	Use in subsequent model simulations
*Spin-up simulation (in addition, or differently, to above)*					
Duration (geomagnetic data)	50 random years, then 25 years 1900	Range 1900–2023	Uniformly (first 50 years)	–	Final-generation used as initial population
Mean natal dispersal	25 m–25 km	Averaged between parents	Initially, uniformly	Evolved	Population-average evolved value used
Intrinsic variability in inheritance of natal dispersal	σ = 2.5 m–1 km	Averaged between parents	Initially, uniformly	Evolved	None (mean natal dispersal is fixed in simulations)
Intrinsic variability in inherited headings	σ = 0.025°–5° (κ = 131–5.3 × 10^6^)	Averaged between parents	Initially, uniformly	Evolved	Population-average evolved value used
Intrinsic variability in inheritance of inclination signposts	σ = 0.1°–1° (κ = 3280–3.3 × 10^5^)	Averaged between parents	Initially, uniformly	Evolved	Population-average evolved value used
Intrinsic variability in inheritance of intensity signposts	σ = 0. 1–1%	Averaged between parents	Initially, uniformly	Evolved	Population-average evolved value used

Top: quantities used in the model simulations (1900–2023), with sensitivity and uncertainty analyses (right column, with all other factors set to defaults). Bottom: additional parameters optimized in initial model spin-up, to derive a viable population, and subsequent use in subsequent simulation (right column). Additional migration-specific parameters (e.g., flight speeds, duration, landing criteria and stopover) are detailed in “Migration model” section and Additional file 2, and data sources in “Model implementation” section

### Evolutionary algorithms

Evolutionary algorithms originated to optimise logistical and workflow problems, through mimicking the iterative process of natural selection of key model parameters [49]. The iterative parameter-evolution approach contrasts with population demographic models based on densities and vital demographic (e.g., birth and death) rates [63]. Many classes and variations of evolutionary algorithms have been developed, including to answer biological questions [50, 51]. A general template for evolutionary algorithms is to first create an initial population of candidate solutions and then, until a given criterion is met, iteratively select a “successful” part of the population to modify for the next iteration (generation), according to the class of evolutionary algorithm used [51]. One such class, evolutionary strategies, combines natural selection and inheritance of traits as real-valued parameters, incorporating both mutations and recombination of traits between iterations [46, 50]. As mentioned, we only retained next-generation (offspring) naïve migrants in the simulation; this is a common technique in evolutionary strategies (known as a comma-strategy as opposed to a plus-strategy [46]). One advantage of evolutionary strategies is in including self-adaptation, i.e., intrinsic variability in inheritance of traits, typically by independently “evolving” a standard deviation in inheritance. We utilised this technique to estimate viable intrinsic variability in inheritance and viable natal dispersal in the *Model spin-up*.

#### Model spin-up and viable population

Prior to each simulation, we performed a model spin-up [18, 52] to evolve viable modelled inherited headings, signposts and natal dispersal, first using geomagnetic data from 50 random years, and then for 25 generations using data from the initial year, 1900. The spin-up process prioritized convergence to a successfully-migrating population over directly identifiable biological dynamics (see Additional file 2 for details). For example, we evolved the extent of intrinsic variability (standard deviation) in inheritance of headings and signposts as individual-level traits (see last 3 rows of Table 1 and Additional file 2). For the subsequent simulations (1900–2023), initial headings and signposts were set to those of the final spin-up population, and intrinsic variability of each trait was conservatively set to its (evolved) population-average value. This is equivalent to assuming that, within the evolutionarily short (124-year) period considered, microevolution of intrinsic variability in inheritance may be constrained, e.g., by limits in plasticity through gene replication and expression [49, 66].

#### Natal dispersal, breeding and trait inheritance

For each departure location, two successful “parents” were randomly selected among all successfully arrived individuals, with selection probability weighted by their natal dispersal distance, *d*, to the focal location [44, 67]. In this way, successful migrants can be seen as a pool of candidate parents, sampled with replacement for each natal location. Natal dispersal was modelled as a half-normal probability distribution with distance [67]; therefore, the probability of selecting a candidate “parent” depended on the ration of *d* to the mean dispersal, ${D}_{N} ($Fig. 2a). During the model spin-up, mean dispersal, ${D}_{N}$, was evolved as an individual trait within a range in means of 25 m–25 km (see Additional file 2). For the resulting viable population, although natal dispersal could be an inheritable trait [44, 67], we chose a more conservative approach by fixing ${D}_{N}$ to be the evolved population average from the spin-up phase. As a sensitivity analysis, we also simulated migration with various (fixed) distributions of natal dispersal, with means ranging from 10-m to 250-km.

### Migration model

We modelled naïve *leucorhoa* wheatears migrating from natal areas in Greenland and North-East Canada (10°–80°W and 57.5°–84°N) to their wintering grounds (hereafter, goal area) in sub-Sahelian West Africa (20°W–5°E and 5°–15°N). To focus on robustness of inherited magnetic-based orientation rather than feasibility of *leucoroha* wheatear migration per se, the model rules were designed to provide both realistic and potentially sufficient compass precision and energy reserves to arrive at the goal area. We here outline the modelled migratory process; natal dispersal and inheritance of orientation traits are described above in “Evolutionary algorithms” section and Fig. 2.

#### Natal locations and initial departure

The same natal locations were used in each simulated year. These were initially set to random point locations with at least 10% low vegetation and less than 15% barren habitat within the surrounding 1° × 1° area, based on EarthEnv Global 1-km Consensus Land Cover [66], producing a distribution closely resembling the known breeding range [60, 68]. We assumed migrants departed on average on August 20th with a 5-day standard deviation, but between August 6th and September 3rd [34, 60].

#### Initial inherited headings and signposts

For the spin-up, initial headings were created based on geographic directions between randomly-chosen natal and (potential) arrival locations, together with offsets for declination for magnetic headings (see Additional file 2 for details). Similarly, initial signposts were set to random values between the magnetic field components at the natal and potential arrival locations on the departure date in 1900 (inclination signposts from von Mises, and intensity signposts from normal distributions). For the actual simulations (1900–2023), initial headings and signposts were set to those of the final spin-up population.

#### Flight steps and identification of signposts

Modelled birds flew at constant 15 m/s ground speeds (accounting for a mean tailwind [34, 69]), following either constant magnetic or geographic (star) compass headings. For magnetic headings, flight directions were updated hourly, accounting for declination changes in spatiotemporal IGRF data [36]. Flight lasted from 90 min after sunset until 90 min before sunrise [68, 70], for minimally 6 h and maximally 12 h, or until land was in sight. We considered a 15° default precision among flight-steps (κ = 14.6), consistent with in-flight measurements of migrating songbirds [71, 72] and model predictions of required precision [16, 19]. With signposted migration, individuals switched headings once on land and when their perceived inclination or intensity fell below the inherited threshold magnitude. We assumed conservatively that, to identify signposts, migrants could gauge magnitudes of inclination with 5° precision [17] and field intensity with 2% precision ([9, 54] i.e., ca. 1000 nT at mid-latitudes). For sensitivity analysis, we assessed migration with 5°–45° directional precision among flight-steps, 0.1°–20° precision in gauging inclination, and 0.1–20% in intensity.

#### Energy reserves and stopovers

Given the initial migratory endurance flight, modelled flight capacities were set to uniformly randomly sampled to between 48 and 72 h, roughly equivalent to 62–106% relative gain in body mass as fat [73], as regularly observed among migrating *leucoroha* wheatears in the wild [57, 69, 73]. Extended migratory stopovers, assumed to last 5 days (14 days at a signpost location), occurred whenever potential flight ranges fell below a threshold (set to three nightly flight durations), or following flight-steps which began at sea [21]. To facilitate crossing the Mediterranean Sea and Sahara Desert [18], modelled energy reserves were replenished at stopovers to provide a potential flight capacity of 48–72 h (sampled uniformly, or as per on arrival if the latter was larger). Refuelling was however not permitted in barren land (0% vegetation or 0% NDVI within 1° × 1° area, 62). If still over water at dawn, modelled individuals stopped at the nearest viewable coastal point (on a ca. 20-km grid), which was identified based on distance to land at each of the last 3 deciles of the nightly flight, with a detection probability which linearly decreased with increasing distance up to a maximum of 300 km (i.e., land immediately on the coast was always detectable, to 50% of the time at 150 km, to never beyond 300 km). If no land was viewable, modelled migrants flew until the next dusk, stopping at the nearest viewable coastal point at each decile of flight, or flying until energy reserves were depleted (mortality).

#### Arrival success

Migrants were considered successful if they arrived in the modelled goal area within a default of 90 days after leaving the breeding area [34, 60]. Signposted migration was still considered successful if migrants arrived in the goal area without having detected a signpost (i.e., when the magnitude of the inherited signpost was lower than that of the relevant geomagnetic component *en route* and on arrival). However, individuals were considered unsuccessful if they overshot the goal area beyond half its latitudinal or longitudinal width (here, 15° in longitude or 5° in latitude), or flew poleward beyond 87.5°N.

### Model implementation

The model was implemented in MATLAB using the parallel programming, statistics and mapping toolboxes. Barren habitat and NDVI were computed using EarthEnv Global 1-km Consensus Land Cover data [62], upscaled to 1° × 1° cells for computation speed. Coastlines were calculated the external Climate Data Toolbox [74], and a MATLAB package adapted for parallel processing of IGRF data [36], updated for the most recent period (2015–2025, [75]). Calculation of topographic (coastline) and geomagnetic cues was performed for all individuals in parallel, as was yearly population mixing (natal dispersal, parent-selection and trait inheritance). Flight durations, ${T}_{fl}$, were calculated using the spherical-Earth formula for solar hour at sunset, *H* (radians), for a given date (solar declination ${\delta }_{s}$) and latitude, $\psi$: $\mathrm{cos}H=\mathrm{tan}\psi \mathrm{tan}{\delta }_{s},$ so that ${T}_{fl}=24\left(1-H/\pi \right)-3$ ([16, 76] this formula is computationally very efficient but ignores slight post-flight latitudinal differences). Regarding computation time, a 75-year (50-year + 25-year) spin-up and 124-year simulation with 50,000 modelled individuals took approximately 5 h to run for hourly-updated magnetic compass in-flight headings on a laptop with an 10th generation Xeon © Intel chip (~ 3 h for star compass in-flight headings).

### Model consistency

To confirm that the default population size (50,000) produced reliable results (evolved orientation and resultant arrival success), we replicated intensity-signposted migration six times for seven different population sizes (between 100 and 250,000). We further confirmed that model results reflected effects of long-term geomagnetic change (e.g., as opposed to lack of convergence in viable orientation) by comparing long-term trends in arrival success to when simulating the 124-year period with geomagnetic data either from a single season (1900) or in reverse chronological order (from 2023 to 1900).

## Results

**Fig. 3:**
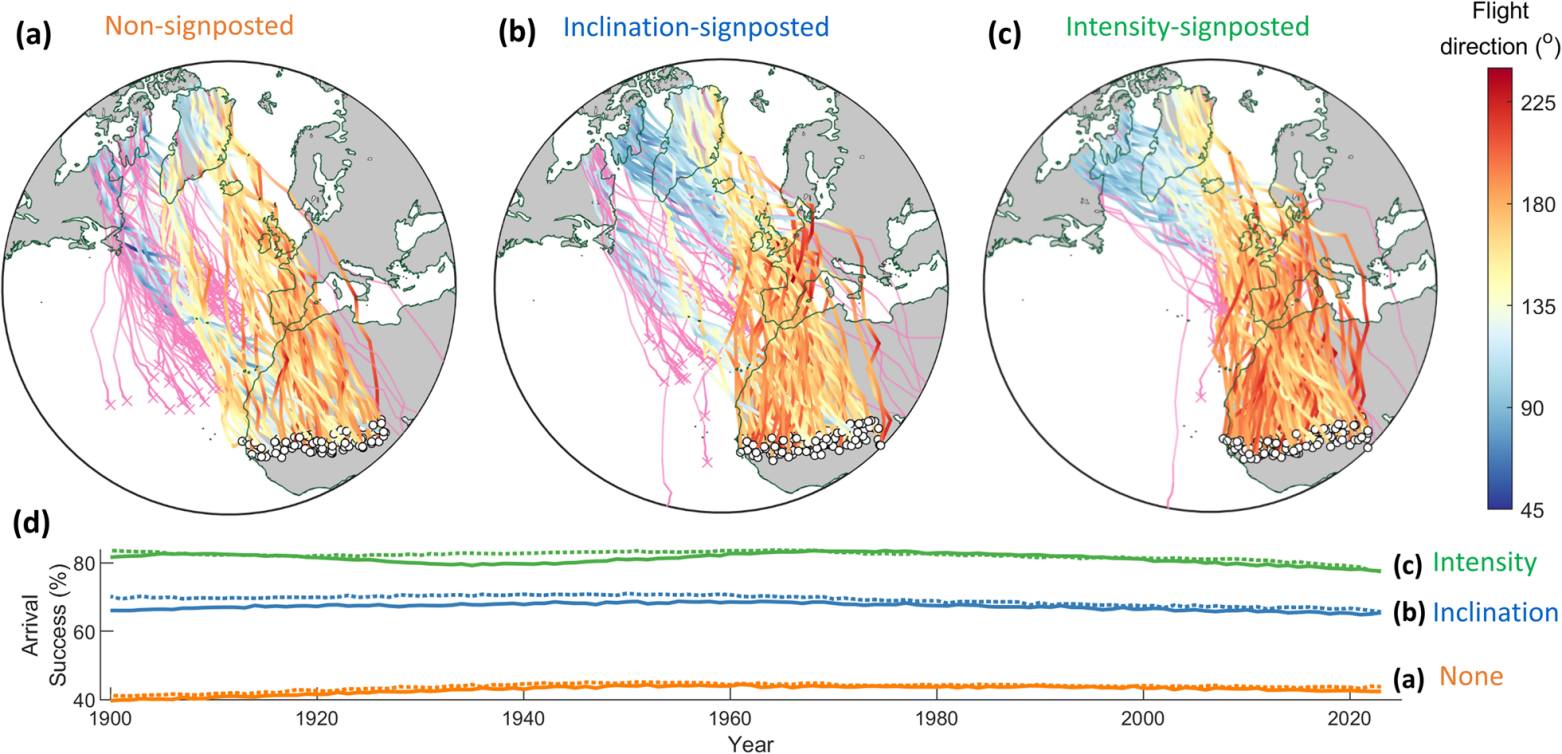
Model-evolved migratory trajectories and arrival success of inexperienced (naïve) *leucorhoa* wheatears (see Fig. 1). Randomly-sampled predicted trajectories from 2023, colour-coded to flight direction (degrees clockwise from geographic N) based on **a** non-signposted migration, following a constant inherited magnetic heading, **b** a magnetic signpost based on inclination and **c** a signpost based on geomagnetic intensity. The results in **a**–**c** are with default model parameters (see “Methods” section and Table 1). For **b**, **c**, encountering a signpost (inherited threshold geomagnetic value) triggers a shift to a second model-evolved inherited heading. Successful arrival in Africa is indicated by white circles, and pink tracks represent unsuccessful individuals. Straight lines represent great circle routes in the stereographic azimuthal projection; **d** arrival success (percentage of population) for non-signposted (solid orange line), inclination-signposted (solid blue line) and intensity-signposted migration (solid green line). Dashed lines depict success when the model is parameterised by geomagnetic data in reverse chronological order (2023–1900)

Model-evolved signposted *leucorhoa* wheatear migration to West Africa was overall and consistently successful across modelled years (1900–2023), with signposted detours over Europe resulting in higher arrival success compared with non-signposted migration. Figure 3 depicts sample modelled trajectories from 2023 for each migratory program based on default model parameters (Table 1). With non-signposted migration (Fig. 3a), less than half of the individuals arrived successfully (43.5 ± 1.2% among years), with frequent over-water mortality (53.8 ± 0.8%) except for shorter ocean crossings such as from Northeast Greenland, or where trajectories came within sight of the Azores, permitting a stopover. With inclination-signposted migration (Fig. 3b), arrival success was higher (67.1 ± 1.1%) and mortality over water more moderate (28.8 ± 1.3%). Intensity-signposted migration (Fig. 3c) almost completely avoided the longest ocean-crossings, resulting in the highest arrival success (79.8 ± 1.5%) and lowest over-water mortality (16.7 ± 1.1%). Nonetheless, success dropped off slightly between 1900 and 2023 with intensity-signposted (2.6%) and inclination-signposted (1.6%) migration, but increased slightly with non-signposted migration (2.7%). Consistent with success being driven by geomagnetic effects, modelled arrival success with geomagnetic data parameterized in reverse chronological order (2023–1900) was slightly higher for all programs (1–2%; dashed lines in Fig. 3d). Reverse-order simulations also reversed the differences in success between 2023 and 1900 (6.1% increase for intensity-signposted, 4.5% increase for inclination-signposted, and 2.5% decrease for non-signposted migration). When using geomagnetic data from 1900 for the entire 124-year simulation, arrival success was ~ 5% higher for signposted migration but ~ 5% lower for non-signposted migration. Results were robust to modelled population size with at least half the default number (25,000 or more) individuals, and also replicable regarding arrival success, mean inherited headings and *Zugknick* latitudes (Additional file 3: Fig. S2). With 10,000 or fewer individuals, between-replicate variability more than doubled regarding arrival success (Additional file 3: Fig. S2a) and evolved headings and *Zugknicks* (Additional file 3: Fig. S2b, c), with success decreasing significantly with 1000 or less modelled individuals.

**Fig. 4:**
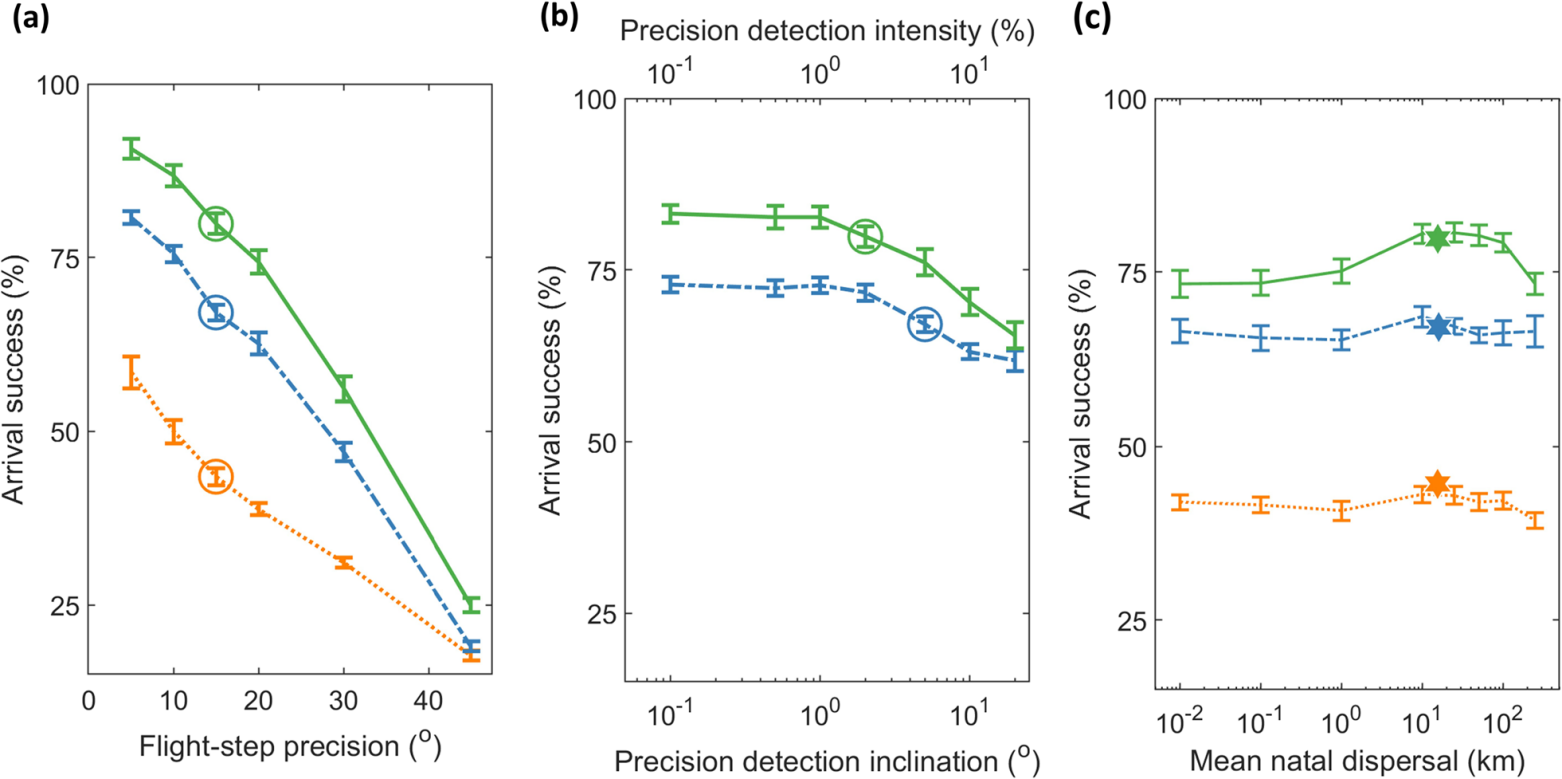
Sensitivity of modelled magnetic-based migration to precision among flight-steps and in gauging signposts. All panels depict long-term mean arrival success (%) and standard deviation among years for non-signposted migration (orange dashed line), and signposted migration based on inclination (dot-dashed blue line) and intensity (solid green line). Arrival success is plotted as a function of **a** precision among flight-steps (degrees), **b** precision in gauging signposts based on inclination (degrees, dot-dashed blue line), and based on intensity (percent intensity, solid green line), and **c** mean natal dispersal (i.e., distance between breeding and natal grounds, km). Circle symbols depict default parameters of **a** 15° flight-flight-step precision and **b** 5° precision in detection of signpost inclination and 2% of intensity signpost. The star symbols in **c** depict (default) model-evolved mean natal dispersal (as in Fig. 3)

For all three orientation programs, arrival success depended strongly on precision among flight-steps (Fig. 4a). While the hierarchy between migratory programs remained consistent (intensity-signposted > inclination-signposted > non-signposted), the difference among them also decreased with lower flight-step precision. Contrastingly, the signposted programs were relatively robust to the degree of precision in gauging inclination or intensity signposts (Fig. 4b). Similarly, each orientation program was relatively robust to the magnitude of mean natal dispersal, up to a mean of 250 km (Fig. 4c). Model-evolved means in natal dispersal (star symbols, Fig. 4c) were ~ 16 km for each default orientation program (as per Fig. 3), resulting in arrival success close to the highest among fixed-distribution simulations.

Overall, magnetic compass use was advantageous in comparison with geographic compass use. Table 2 compares, for default compass precision (circles in Fig. 4a, b), mean arrival success for all combinations of magnetic and geographic compass use, for both signposted and non-signposted migration. Once again, the hierarchy in performance among non-signposted and signposted migration remained the same, with declination-signposted programs less successful compared with other signposted programs (52% vs. 67–80% arrival success, with all-magnetic compass use). Inheriting magnetic as opposed to geographic headings benefitted arrival success among signposted programs (median 15.9% relative gain in success, range 8.4–40.0%), but not so with non-signposted migration (median − 0.6% relative gain, range − 2.5 to 0.4%). Contrastingly, non-signposted migration with a primary magnetic compass always performed better (median 47.6%, range 43.1–54.8%) than with a primary star compass, but among signposted programs this effect was weaker (median 3.9%, range − 1.9 to 31.8%). Finally, using a geographic (star) in-flight compass clearly increased performance of geographically-inherited programs with an inclination compass (median 15.0%, range 11.9–18.1%) but had no clear or consistent effect among other signposted programs (median 3.2%, range − 3.3 to 13.6%), nor for non-signposted programs (median 0.8%, range − 2.8 to 2.6%). Taken together, it is interesting that for both non-signposted and signposted programs, purely magnetic compass use (top row) consistently outperformed purely geographic compass use (bottom row; median 19.8%, range 9.3–42.2%).

**Table 2:** Arrival success among choices in compass use, for non-signposted and signposted migration

Inherited headings	Primary compass	In-flight compass	Non-signposted	Inclination-signposted	Intensity-signposted	Declination-signposted
Magnetic	Magnetic	Magnetic	43.5 ± 1.2%	67.1 ± 1.1%	79.8 ± 1.5%	52.4 ± 2.3%
		Geographic (star)	43.5 ± 0.6%	70.2 ± 1.9%	**82.2** ± **2.3**%	**52.7** ± **1.7**%
	Geographic (star)	Magnetic	28.9 ± 0.5%	68.4 ± 2.9%	78.8 ± 2.7%	43.7 ± 3.3%
		Geographic (star)	28.1 ± 0.3%	**70.9** ± **3.6**%	80.6 ± 3.8%	46.0 ± 3.3%
Geographic	Magnetic	Magnetic	43.1 ± 1.3%	53.2 ± 3.4%	72.9 ± 1.6%	47.7 ± 2.1%
		Geographic (star)	**43.8** ± **1.1**%	60.4 ± 2.1%	70.6 ± 1.3%	48.6 ± 2.6%
	Geographic (star)	Magnetic	29.8 ± 0.4%	50.3 ± 1.7%	64.7 ± 1.0%	36.2 ± 2.1%
		Geographic (star)	30.6 ± 0.2%	61.4 ± 1.3%	67.8 ± 0.9%	41.9 ± 0.9%

Arrival success (geometric-mean ± between-year standard deviation) for migratory orientation programs for all combinations of geographic (star) compass and magnetic compass use, i.e., regarding inherited headings, primary migratory compass (used to re-determine headings on departures), and in-flight compass (used to re-determine hourly flight headings). In addition to inclination and intensity signposts (Figs. 3, 4), declination-signposted migration is also listed (last column). The first row lists the default model, with all-magnetic compass use. The highest performance for each program (e.g., inclination-signposted) is listed in bold. In all cases, other parameters were set to default values (see Table 1)

**Fig. 5:**
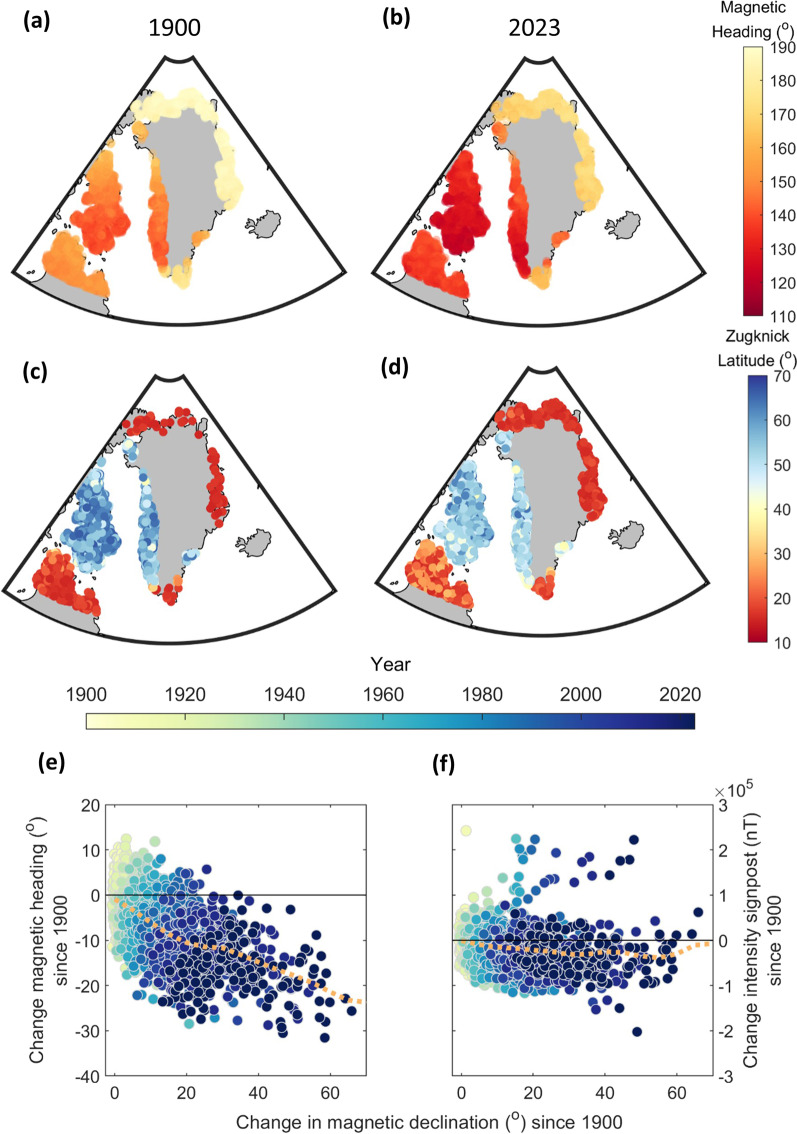
Evolution of modelled intensity-signposted migration of *leucoroha* wheatears to geomagnetic secular variation. Coloured symbols of 5000 randomly-selected successful modelled individuals illustrate **a**, **b** inherited magnetic headings (clockwise degrees from magnetic N) and **c, d** *Zugknick* latitudes (degrees), from 1900 (**a**, **c**) and 2023 (**b**, **d**). Yearly changes relative to 1900 regarding **e** initial inherited headings (° clockwise) and **f** intensity signposts (nT) among randomly-selected individuals (natal locations), as a function of change in declination at the natal site (° clockwise) since 1900, colour-coded per year (scale on top). Orange dashed lines represent mean changes in **e** headings and **f** intensity signposts (nT), sorted in 5° bins of declination change. **a**–**d** Stereographic azimuthal projection

Both signposted programs evolved a sharp migratory divide in trans-Atlantic routes, and underwent relatively stronger temporal shifts in migratory headings than signposts over the 124-year period. Figure 5 illustrates this regarding headings (Fig. 5a, b) and *Zugknick* latitudes (Fig. 5c, d) for intensity-signposted migration. Individuals breeding in NE and N Greenland evolved close to magnetic S headings (~ 180°) with *Zugknicks* in Africa, whereas individuals breeding in Canada and S and W Greenland, evolved closer to magnetic SW headings (~ 135°) with *Zugknicks* in W Europe. In response to the clockwise (positive) drift in declination, magnetic headings shifted counter-clockwise between 1900 and 2023 (Fig. 5e, mean − 16.5°), and *Zugknick* signposts decreased (Fig. 5f, mean − 380 nT, or − 0.7%), resulting in a Southward latitudinal shift in *Zugknicks* (Fig. 5d, mean − 5.2°). Inclination-signposted migration (Additional file 4: Fig. S3) evolved a similarly SW-NE contrast in headings and shift in headings (mean − 16.2°) and signposts (mean − 3.2°), but individuals breeding in N Quebec and the Southern tip of Greenland evolved more direct (though less successful) routes towards Africa rather than via Europe. Long-term shifts in headings and routes, and their effect on arrival success varied over time and regionally: Additional file 4 (Fig. S4) details for migration from Baffin Island (60°–80°W, 62.5°–70°N) how these shifts accelerated in the second half of the study period (1961–2023), resulting in increased (~ 10% higher) over-water mortality for this Western fringe of the population.

**Fig. 6:**
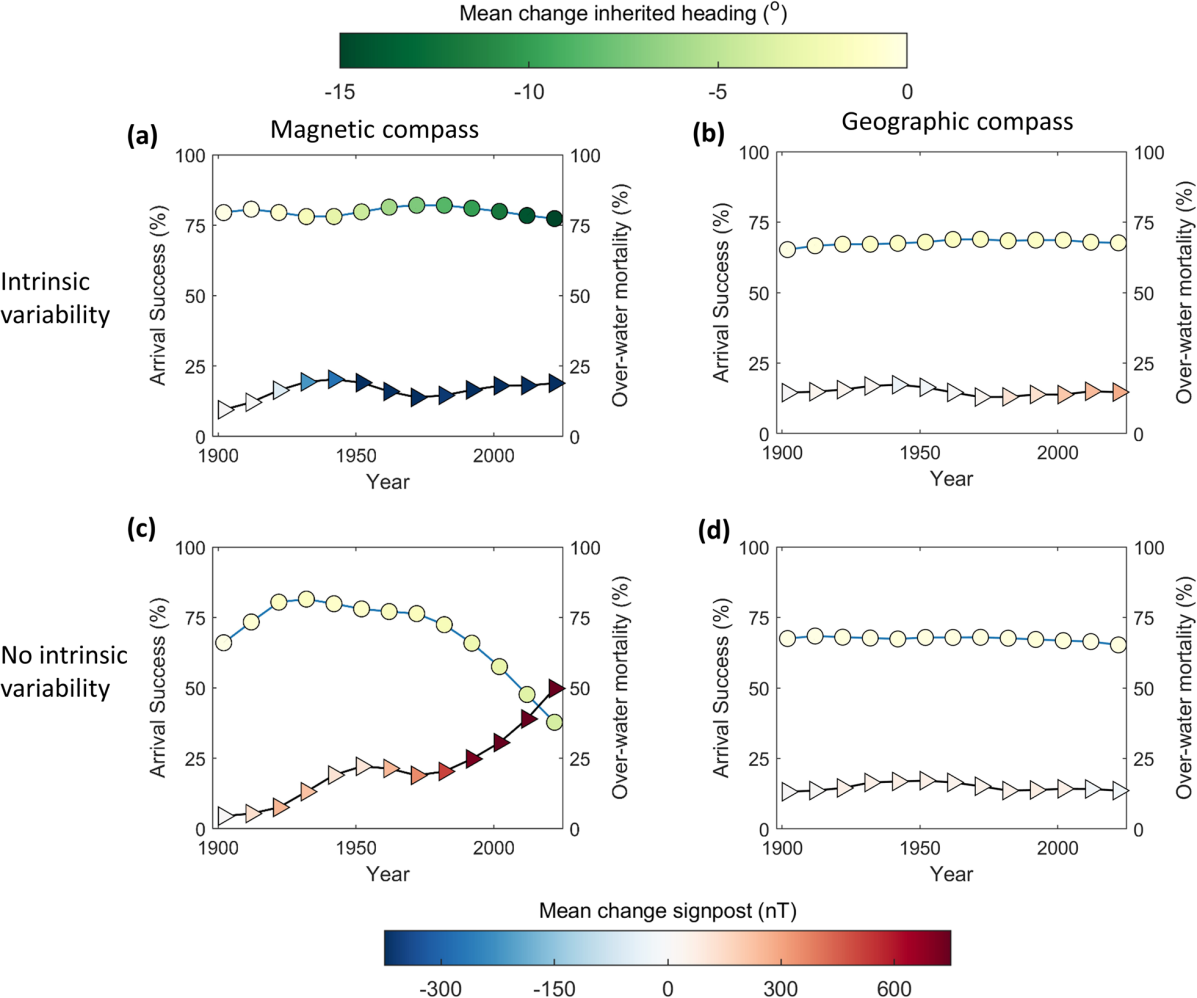
Intrinsic variability in inheritance facilitates robustness of magnetic headings and signposts to geomagnetic secular variation. Circles represent mean successful arrival (left axes) and triangles mortality over water (right axes) among intensity-signposted migrants (Fig. 3c), with symbol colours depicting population-mean changes in inherited headings since 1900 (circles, clockwise degrees from magnetic N) and in signpost magnitude (triangles, nT). **a** With model-evolved standard deviation in inherited headings (2.6°) and intensity signposts (0.53%); **b** as **a** but with an inherited geographic (star) primary and in-flight compass; **c** is as **a** and **d** as in **b**, but without intrinsic standard deviations in inheritance of headings and signposts

Plasticity in inherited magnetic headings and robustness to the strong secular variation was contingent on inheritance being intrinsically variable, as illustrated in Fig. 6 for intensity-signposted migration. Figure 6a illustrates, for inheritance involving intrinsic variability, population-mean changes in inherited magnetic headings (circle colours) and intensity signposts (triangle colours); this resulted in consistent arrival success (~ 80%, left axis) and limited over-water mortality (< 25%, right axis). However, with perfect inheritance (zero standard deviations, Fig. 6b), headings failed to adapt (mean shift − 0.2° vs. − 16.5°) while signposts increased, reducing arrival success to 29% in 2023 as over-water flights became unsustainable (> 50% mortality). With geographic-inherited headings and compass use (but with *Zugknicks* still triggered by magnetic intensity signposts, Fig. 6c, d), arrival success was overall lower either with (68%, Fig. 6c) or without intrinsic variability (67%, Fig. 6d). With a primary geographic compass, inherited headings (circles, Fig. 6c, d) are not expected to vary strongly with secular magnetic change, but intensity signposts (triangles, Fig. 6c, d) also shifted much less compared with magnetic inherited headings (Fig. 6a, b). Model-evolved standard deviations in inheritance of headings were consistent across orientation programs, ranging from 2.3° to 2.7° among non-signposted and non-signposted programs, including across the range of tested distributions of natal dispersal (Fig. 3c), and also with smaller population sizes (Fig. 3b). Model-evolved standard deviations in signposts were 0.58° for default inclination-signposted and 0.53% for default intensity-signposted programs, and in the sensitivity analysis were similar across all tested distributions of natal dispersal (0.54 ± 0.2° and 0.54 ± 0.03%).

## Discussion

This study represents a first assessment of how migratory populations can adapt to complex and drifting geomagnetic landscapes, through natural selection of inherited magnetic information, together with a primary magnetic migratory compass. In particular, model results support the idea that inexperienced migrants can negotiate detoured routes using inherited magnetic headings and signposts. Such a gauge-and-compass program, where a magnetic “gauge” rather than circannual clock triggers switches in compass headings, could be particularly important for populations where naïve migrants travel independently, for example in providing a mechanism to evolve novel routes shaped by shifting habitat suitability and climate refugia [23, 77, 78]. Magnetic signposts can in principle also trigger orientation shifts between celestial compass headings, such as a sunset compass or star compass [15, 16]. Depending on inner clock-updates and sensitivity to variable scheduling, sun-compass headings may be particularly advantageous over long-distance and high-latitude routes [16].

For *leucorhoa* wheatears, it is unsurprising that *Zugknicks* via Europe are of adaptive benefit, given the risk of insufficient energy reserves for the minimally 4000-km direct open-ocean flights to Africa [20, 40]. Nonetheless, the persistent success of modelled magnetic-based migration (Figs. 3, 6a) is somewhat remarkable, illustrating that strongly varying geomagnetic landscapes can be advantageous rather than necessarily represent a hazard. The fact that inherited magnetic headings and a primary magnetic compass generally outperformed their geographic counterparts (Table 2, Fig. 6) probably relates to the clockwise spatial gradient in declination (Fig. 1) which reduces the ocean crossing [15, 35] and facilitates self-correction in orientation (Additional file 1: Fig. S1). The relative favourability of a primary magnetic over other compasses will however additionally be contingent on available mechanisms [54], cue reliability [9, 39] and relative compass precision [16]. Magnetic disturbances including geomagnetic storms typically have little effect on compass precision (< 2° shifts in declination, 9), unlike with gradient-based navigation [9, 38]. Naïve migrants could nonetheless be affected by geomagnetic storms at high latitudes [27, 33], where determination of the N–S geomagnetic axis in near-vertical fields can be challenging [5, 11, 17].

The counter-clockwise shift in inherited magnetic headings over the 124-year period (Figs. 5, 6) is expected given the clockwise geomagnetic drift on the natal grounds (Fig. 1). Route geometry and the risky ocean crossing may have constrained *Zugknick* signposts and locations much more strongly compared with headings (Figs. 5, 6, Additional file 4, Fig. S3), but inclination and intensity in W Europe also varied less strongly compared with declination over the 124-year period (Additional file 5, Fig. S5). It is further interesting that both inclination-signposted and intensity-signposted modelled populations evolved sharp SW-NE migratory divides, with the NE subpopulation (NE Greenland) almost not requiring a *Zugknick* to reach West Africa (Fig. 5, Additional file 4: Fig. S3). Such a scenario could lead to effective reproductive isolation through hybrid mortality [79], or alternatively development of dominance inheritance patterns between competing alleles [43], in contrast to the co-dominance (averaging) of traits modelled here. Naturally, the actual routes taken by *leucoroha* wheatears will be modulated by other factors not fully considered, such as capacity for migratory endurance flight [40, 73], reliability of selected winds [19, 24] and avoidance of hazards associated with the longer detour, e.g., diminishing seasonal resources or exposure to predation [20]. Our sensitivity analysis to population size (Additional file 3: Fig. S2) illustrates more generally that if arrival success and population sizes become very low, population mixing can no longer track the strong secular geomagnetic change; this can be seen as a sort of Allee effect, particularly noticeable in fringe populations (Additional file 4: Fig. S4), analogous to loss of migratory connectivity under habitat loss [80].

Intensity-signposted programs outperformed inclination-signposted programs along this route, possibly related to more advantageous spatial gradients (Additional file 5: Fig. S6), enabling more Easterly routes with reduced risk of over-water mortality or overshooting the wintering area (Additional file 4: Fig. S4). However, favourability among potential signposts will depend on the exact nature of the avian magnetic compass mechanism, which remains uncertain. Magnetic inclination is as mentioned discernible to naïve migrants, and with the favoured radical-pair magnetoreception, geomagnetic intensity will amplify the received signal [81]. Therefore, it seems reasonable to conjecture that inexperienced migrants might be able to “gauge” a magnetic signal based on intensity, inclination or some combination of both [82]. Gauging magnetic declination seems less likely for naïve migrants, since it requires comparison of geographic and geomagnetic axes, often while on the move and close to dark [16, 83]. Declination signposts additionally underperformed for modelled *leucoroha* wheatears, and were not sufficient for experienced migrants to correct for displacement in a recent experiment [7].

Lastly, our results support the idea that intrinsic variability in inheritance of migratory orientation, in addition to facilitating expansion of breeding and non-breeding ranges [12, 47, 84], is important in maintaining or modulating routes in unpredictable environments [42]. Including such variability, arrival success decreased by only ~ 3% (Fig. 6a) in the rapid secular variation in the second half of the study period (mean shift 23° vs. 11°, Fig. 1), as opposed to > 40% when not included (Fig. 6b). The extent to which intrinsic variability in inheritance would either be evolvable through natural selection or constrained by molecular (genetic or developmental) processes remains unknown.

It is important to consider why inexperienced migrants might use a magnetic signpost to mediate detours rather than migratory cues such as habitat quality [22] or topography [85]. While clearly important, the latter extrinsic cues may not be sufficiently unambiguous in negotiating long-distance routes. For example, an inherited *Zugknick* to reorient to the South on completing an open-ocean endurance flight could work for *leucoroha* wheatears arriving to Western Europe but, if they first stopped in Iceland, additional information would be required to avoid misorientation into the mid-Atlantic Ocean. We therefore propose that naïve migratory orientation responses to coastal and habitat cues could be mediated or triggered by magnetic information during the long-distance phase of migration, similarly to with energetic and stopover decisions [86]. Given that magnetic declination and celestial cues are fairly stable within migratory periods, we speculate that inherited compass information is typically primary, at least among long-distance nocturnally-migrating birds [5, 16], though the degrees to which other environmental and social cues refine and modulate this programme remain to be clarified [3, 87–89].

As an alternative to compass-based inherited orientation, it has also been proposed that naïve migrants might be able to perform navigation by following gradients in the geomagnetic field [8]. The relative feasibility and efficiency of constant-heading versus gradient-based migration remains an open question [9, 16, 38]. For actual migration systems, this has only been assessed for migration based on correlated random walks—per definition less directed than compass courses—with supplemental navigational abilities based on geomagnetic information [38]. While gradient-based navigation using innate or early-learned information offers the possibility to correct for imprecision or displacement [88], e.g., by currents, it could also produce inefficient migrations when gradients in field components are closely aligned [9, 29, 38, 90]. Our results suggest that constant-heading migration modulated by magnetic signposts could be sufficiently robust to variable and changing geomagnetic fields, at least given suitable compass precision and intrinsic variability in inherited headings.

## Conclusions

While global patterns of avian migration can be explained as efficient energy acquisition of seasonal resources [91], enabling hindcasts of prehistoric migration routes [22, 77], little is understood regarding the population consequences of how migratory orientation is transmitted across generations [43, 88, 89]. Using an evolutionary algorithm approach enables population-level assessments of how inherited migratory orientation programs can both mediate and constrain adaptation of historic and novel migration routes. Our methods can be extended to assess other geophysical cues (e.g., sun azimuth) and flexible orientation reactions to other environmental factors such as currents, coastlines and habitat quality [24, 69, 85], including to assess resilience to climate change. More generally, our results illustrate how the Earth’s magnetic field may possibly play a vital role in the evolution of migration routes, as mediator between proximate environmental cues and ultimate drivers of population fitness through migratory success.

## Supplementary Information

**Additional file 1**. **Fig. S1**. Positive longitudinal gradients in geomagnetic declination facilitate self-correction using a geomagnetic compass.

**Additional file 2**. Additional details of model spin-up and initialisation.

**Additional file 3**. **Fig. S2**. Validation of model consistency with number of modelled individuals, for intensity-signposted migration.

**Additional file 4**. **Fig. S3**. Evolution of modelled inclination-signposted migration of *leucoroha* wheatears to long-term geomagnetic shifts. **Fig. S4** Secular changes in geomagnetic headings, *Zugknicks* and arrival locations among modelled intensity-signposted *leucoroha* wheatears.

**Additional file 5**. **Fig. S5**. Changes in geomagnetic inclination and intensity between 1900 and 2023. **Fig. S6**. Contours in geomagnetic inclination and intensity between 1900 and 2023.

## Data Availability

The code to simulate the model and reproduce all the results figures is available in github repository (https://github.com/jdmclaren/evo_migration).

## Abbreviations

N: North
S: South
E: East
W: West
