# Supplementary material for: Gauge-and-compass migration: inherited magnetic headings and signposts can adapt to changing geomagnetic landscapes
Source: Mov Ecol. 2023 Jul 5;11:37. doi: 10.1186/s40462-023-00406-0 (PMC10320893; doi:10.1186/s40462-023-00406-0)
Supplement: Supplementary file 1 — Additional file 1. Fig. S1. Positive longitudinal gradients in geomagnetic declination facilitate self-correction using a geomagnetic compass. [file 40462_2023_406_MOESM1_ESM.docx]

| 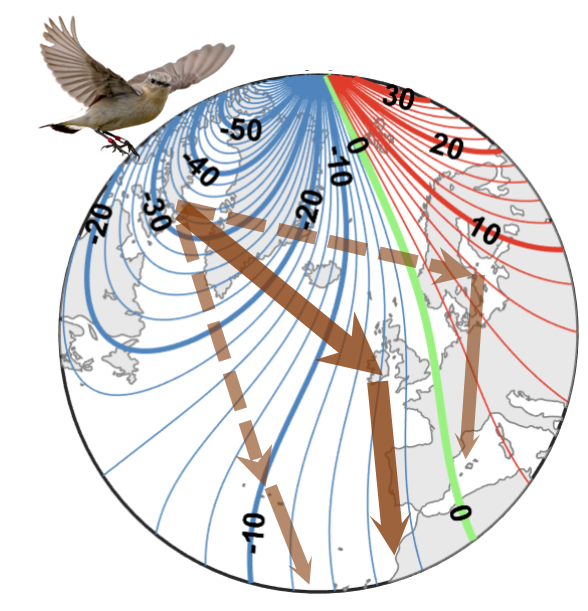 | **Fig. S1** Positive longitudinal gradients in geomagnetic declination facilitate self-correction using a geomagnetic compass. Contours of declination (clockwise degrees between true geographic and magnetic N) for 2010, together with likely routes taken by *leucoroha* wheatears (photo, HS). Magnetic data are from a global IGRF modelled data of the Earth’s core-field (1,2). Solid thick brown arrows represent approximate route taken from Baffin Island, Canada and West Africa (see Fig. 1, main text). If displaced *en route* (dashed brown arrows), the resultant *Zugknick* flight direction (on reaching the threshold intensity or inclination; thin solid brown arrows) partially compensates for the displacement, due to the counter-clockwise declination shift when displaced Westward, and clockwise shift when displaced Eastward. |
| --- | --- |

References

1. Compston D. International Geomagnetic Reference Field (IGRF) Model [Internet]. 2022. Available from: https://www.mathworks.com/matlabcentral/fileexchange/34388-international-geomagnetic-reference-field-igrf-model

2. Thébault E, Finlay CC, Beggan CD, Alken P, Aubert J, Barrois O, et al. International Geomagnetic Reference Field: the 12th generation. Earth Planet Sp. 2015 Dec;67(1):79.
