# Supplementary material for: Gauge-and-compass migration: inherited magnetic headings and signposts can adapt to changing geomagnetic landscapes
Source: Mov Ecol. 2023 Jul 5;11:37. doi: 10.1186/s40462-023-00406-0 (PMC10320893; doi:10.1186/s40462-023-00406-0)
Supplement: Supplementary file 2 — Additional file 2. Additional details of model spin-up and initialisation. [file 40462_2023_406_MOESM2_ESM.docx]

**Additional File 2: Details of model spin-up and initialisation**

For the model spin-up, inherited headings, signposts and (mean) natal dispersal were chosen to provide sufficient variability to converge to a viable (successful) population, together with iterative selection and trait mixing. Unlike in the full model simulations (1900-2023), this mixing was not as directly interpretable in biological terms regarding trait inheritance.

As mentioned in the main text, in addition to headings and signposts being evolved from successful migrants (see Fig. 2), intrinsic variability and mean dispersal were also “passed on” during the spin-up phase (intrinsic variability in headings and inclination signposts were based on von Mises concentrations, κ, and intensity signposts based on standard deviations, σ, in percentage of total field intensity). Moreover, inheritance of intrinsic variability and mean natal dispersal was also intrinsically variable, as described below (and listed in the lower half of Table 1). Evolution of intrinsic variability and mean natal dispersal was performed only in the first phase of the spin-up (using magnetic data from 50 random years). In the second spin-up phase (simulating 25 migratory generations with geomagnetic data from the initial year), we conservatively used constant (population-uniform) intrinsic variability and mean natal dispersal, just like in the full simulations. This allowed the population to adapt headings and signposts to these uniform traits, as well as to the geomagnetic landscape in 1900.

*Initialising orientation traits*

Initial magnetic headings and *Zugknick* headings were created from a pool of headings centred on the means of loxodromic (constant geographic heading) and great-circle headings between each natal and potential (randomly-chosen) arrival locations, together with a random offset, and an offset for initial magnetic declination in 1900 (the latter offset only for magnetic inherited headings). The random offset was drawn from a von Mises distribution (κ = 0.033, ~17.5° standard circular deviation), constrained within -180° to 180°. Headings and also *Zugknick* headings for each natal location were chosen randomly from this pool, resulting in magnetic W to S initial headings (95% within 87°-182° clockwise from magnetic N, corresponding to 41°-151° from geographic N). During the first (50-year) spin-up phase, intrinsic variability in headings was initially populated to uniformly sampled values between κ = 131 – 5.3∙10^6^ (σ = 0.025° – 5°), in inclination signposts between κ = 3280 – 3.3∙10^5^ (σ = 0.1° – 1°) and in intensity signposts between σ = 0. 1 – 1%.

*Initialising natal dispersal*

We similarly evolved mean dispersal, $D_{N}$, as an individual trait during the first (50-year) spin-up phase. Mean dispersal was initially populated uniformly between 25-m and 25-km, and intrinsic variability (standard deviation) in inheritance of mean dispersal was initially uniformly distributed between 2.5-m and 1-km. For the second (25-year) spin-up phase and subsequent simulation (1900-2023), $D_{N}$ was set to its population average. For the sensitivity analysis, we also simulated migration with various population-uniform (fixed) values of mean natal dispersal, ranging from 10-m to 250-km. For these simulations, no evolution of $D_{N}$ was performed during model spin-up.

*Acceleration of convergence*

For computational efficiency in convergence during the first spin-up phase, we additionally weighted selection of the second candidate parental migrant by its similarity in intrinsic variability, as a form of assortative mating (this is known as self-adaptation in Evolutionary Strategy algorithms; 1,2). That is, weights of the second chosen parent were multiplied by the relative differences in standard deviations compared with the first parent regarding inheritance of headings, signposts and regarding mean dispersal, with zero weights when maximally different and a maximal weight of one when identical.

References

1. Rudolph G. Evolutionary Strategies. In: Rozenberg G, Bäck T, Kok JN, editors. Handbook of Natural Computing [Internet]. Berlin, Heidelberg: Springer; 2012 [cited 2022 Aug 4]. p. 673–98.

2. Emmerich M, Shir OM, Wang H. Evolution Strategies. In: Martí R, Panos P, Resende MGC, editors. Handbook of Heuristics [Internet]. Cham: Springer International Publishing; 2018
