# Supplementary material for: Gauge-and-compass migration: inherited magnetic headings and signposts can adapt to changing geomagnetic landscapes
Source: Mov Ecol. 2023 Jul 5;11:37. doi: 10.1186/s40462-023-00406-0 (PMC10320893; doi:10.1186/s40462-023-00406-0)
Supplement: Supplementary file 3 — Additional file 3. Fig. S2. Validation of model consistency with number of modelled individuals, for intensity-signposted migration. [file 40462_2023_406_MOESM3_ESM.docx]

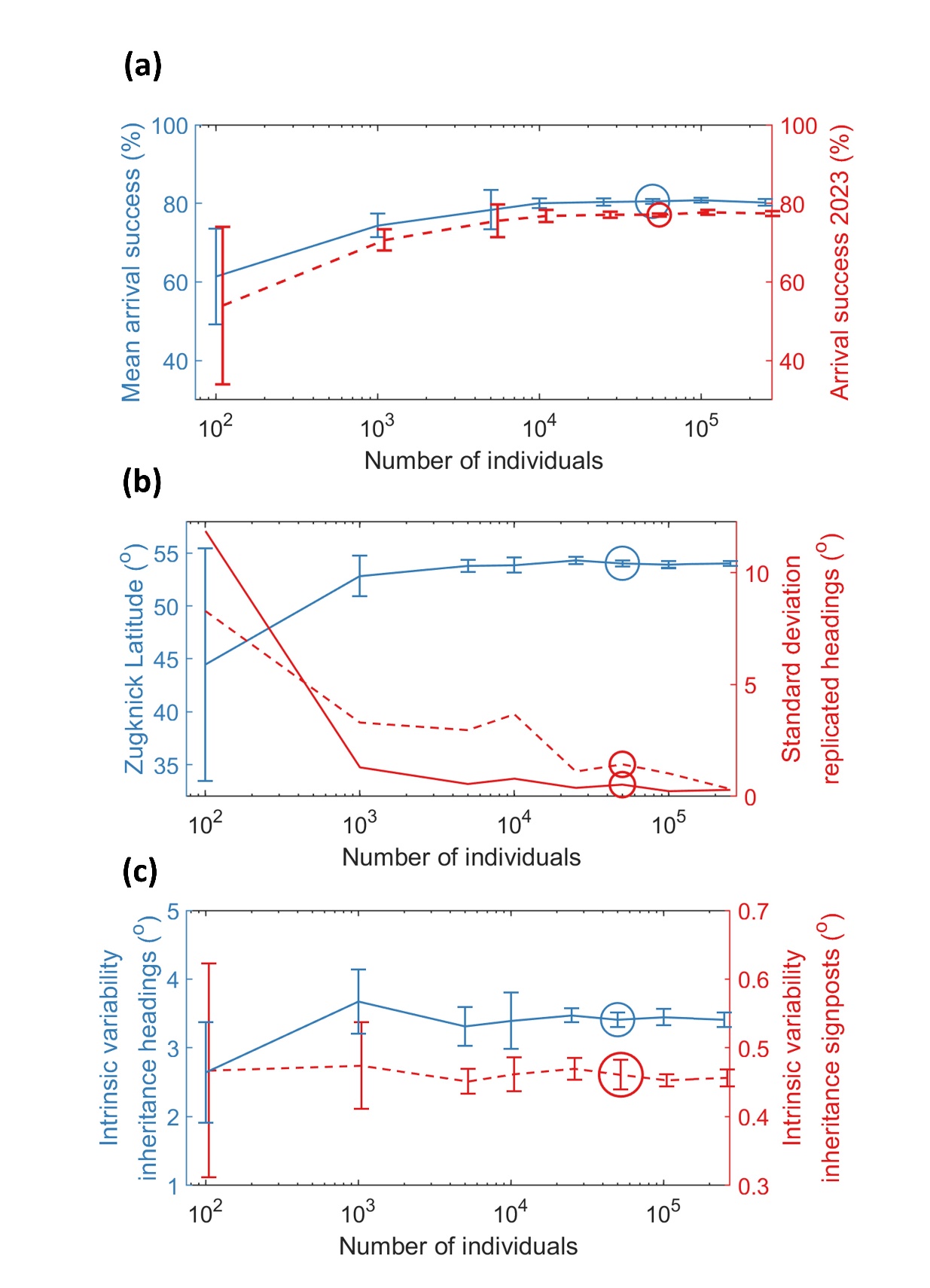


**Fig. S2** Validation of model consistency with number of modelled individuals, using 6 replicates for intensity-signposted migration. (**a**) geometric-mean arrival success (blue lines) and final-year success (dashed red line) with, here and elsewhere, error bars depicting standard deviation over replicates; (**b**) population-mean final-year *Zugknick* latitude (blue lines and error bars) and between-replicate standard deviation in population-mean final-year inherited headings (red lines) and *Zugknick* headings (dashed red lines), all in degrees, (**c**) as evolved in the model spin-up, intrinsic standard deviation in inheritance of migratory headings (degrees, blue lines and error bars) and inheritance of intensity-signposts (% of actual field concentration, red lines and error bars).
