# Supplementary material for: Gauge-and-compass migration: inherited magnetic headings and signposts can adapt to changing geomagnetic landscapes
Source: Mov Ecol. 2023 Jul 5;11:37. doi: 10.1186/s40462-023-00406-0 (PMC10320893; doi:10.1186/s40462-023-00406-0)
Supplement: Supplementary file 4 — Additional file 4. Fig. S3. Evolution of modelled inclination-signposted migration of leucoroha wheatears to long-term geomagnetic shifts. Fig. S4 Secular changes in geomagnetic headings, Zugknicks and arrival locations among modelled intensity-signposted leucoroha wheatears. [file 40462_2023_406_MOESM4_ESM.docx]

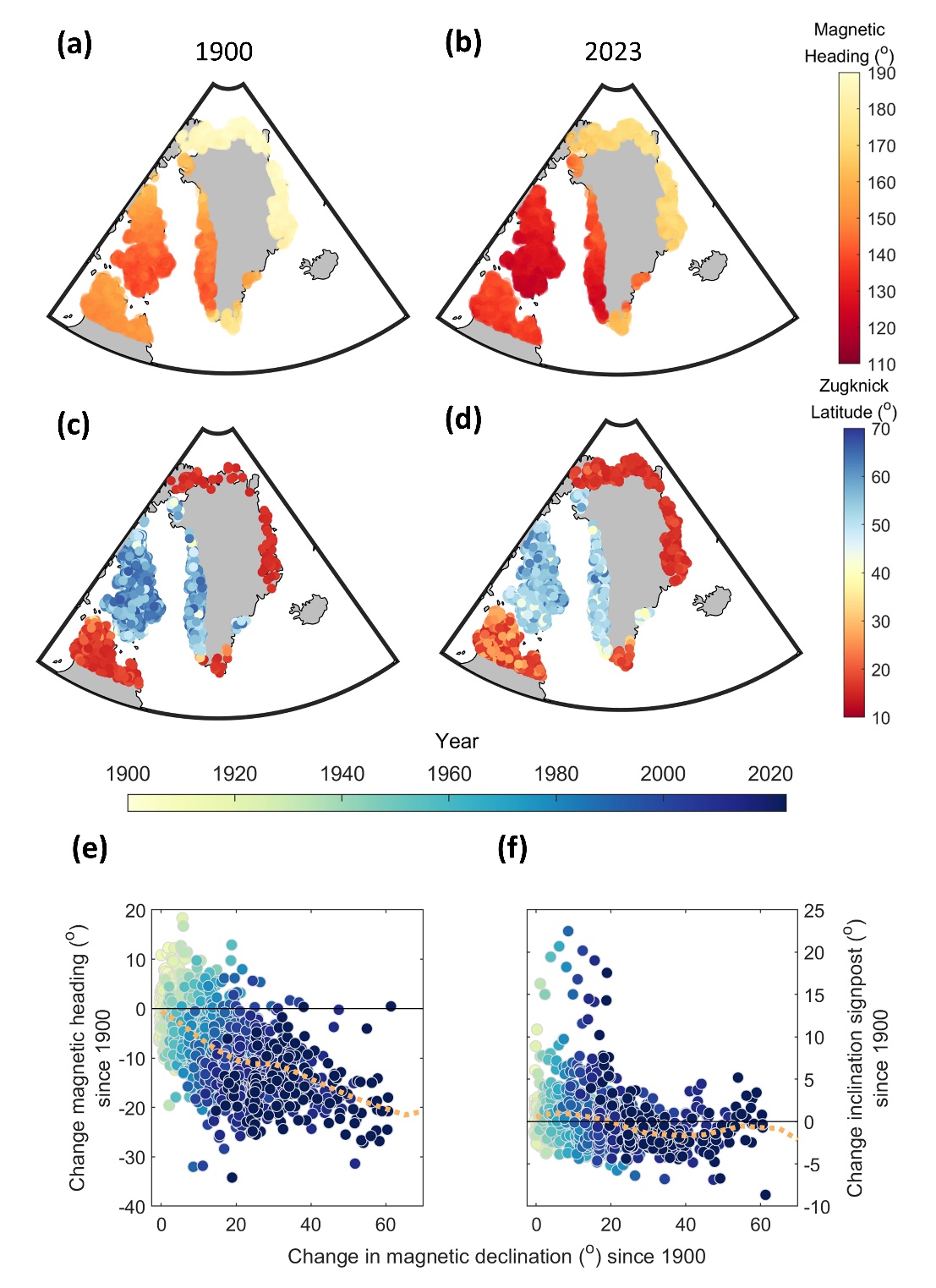


**Fig. S3** Evolution of modelled inclination-signposted migration of *leucoroha* wheatears to long-term geomagnetic shifts. As in Fig. 5, but for inclination-signposted migration. Coloured symbols represent 5000 randomly-sampled inherited geomagnetic headings (**a**) and *Zugknick* latitude (**b**) in 1900, illustrate a SW/NE divide in headings but three-way division in migratory connectivity (individuals breeding in the NE and S/SW evolving direct routes to Africa, and individuals breeding in the W detoured routes. In 2023, (**c**) inherited magnetic headings shifted clockwise (mean -16.2°) while (**d**) *Zugknicks* underwent small Southward shifts (mean -3.2°). Yearly changes relative to 1900 regarding (**e**) initial inherited headings and (**f**) inclination signposts (both ° clockwise) among randomly-selected individuals (natal locations), as a function of change in declination (° clockwise) since 1900, colour-coded per year (scale on top). Orange lines represent mean changes in (e) headings and (f) signposts (maximally 0.5° in 1978), sorted in 5° bins of declination change. Stereographic azimuthal projection.


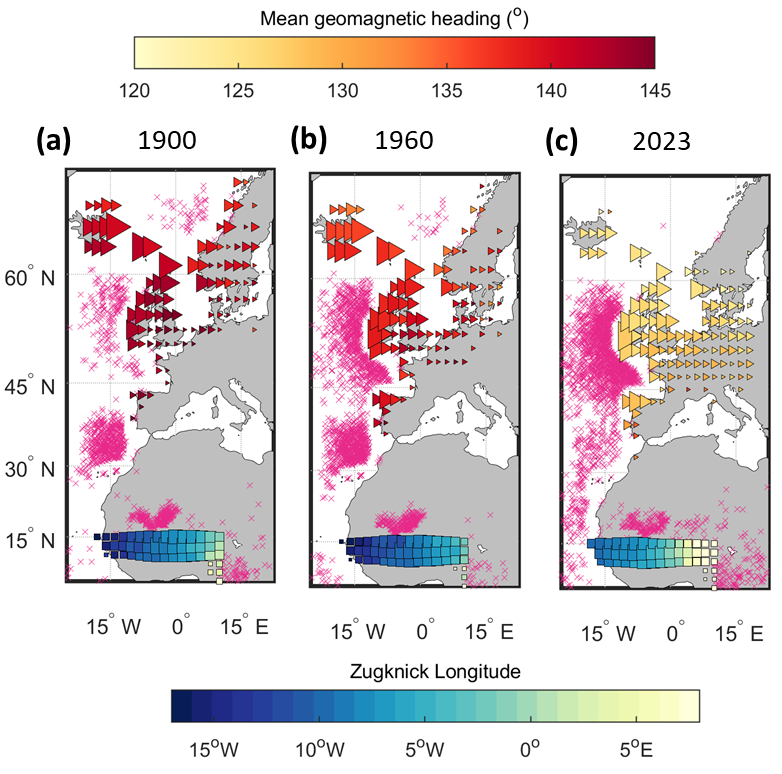


**Fig. S4** Secular changes in geomagnetic headings, *Zugknicks* and arrival locations among modelled intensity-signposted *leucoroha* wheatears, between (**a**) 1900, (**b**) 1960 and (**c**) 2023, among modelled intensity-signposted *leucoroha* wheatears (main text, Fig. 3a). For visual clarity we focused on modelled individuals from Baffin Island (60°-80°W, 62.5°-70°N). Triangles depict locations of *Zugknicks* and overlapping squares depict arrival points at the wintering grounds. Symbol sizes are proportional to frequency of occurrence within 2°x2° areas, with triangle colours depicting mean inherited headings (clockwise degrees from magnetic N) and square colours mean *Zugknick* longitude. Pink crosses depict mortality over water, desert or having overshot the arrival area to the South or East.

In 1900, *Zugknicks* occurred most frequently between Iceland and Scotland (triangles in Fig. S4a), following fairly uniform magnetic headings, with broad-front arrival to Africa (squares, coloured by *Zugknick* longitude with size scaled by frequency). By 1960 (Fig. S4b), *Zugknicks* were shifted slightly towards Ireland (mean 2.2° Southward and 1.9° Westward shifts), with mean magnetic headings shifted 5.1° counter-clockwise (NW) but mean initial departure directions (geographic headings) shifted 8.6° clockwise (SE), resulting in 6.5% higher over-water mortality (pink crosses). By 2023 (Fig. S4c), magnetic headings were shifted farther counter-clockwise (15.9°) yet initial geographic headings farther clockwise (23.5°), resulting in more continental *Zugknick* locations (mean 3.2° Eastward and 7.3° Southward shifts), and 9.8% higher over-water mortality compared with 1900.
