# Supplementary material for: Gauge-and-compass migration: inherited magnetic headings and signposts can adapt to changing geomagnetic landscapes
Source: Mov Ecol. 2023 Jul 5;11:37. doi: 10.1186/s40462-023-00406-0 (PMC10320893; doi:10.1186/s40462-023-00406-0)
Supplement: Supplementary file 5 — Additional file 5. Fig. S5. Changes in geomagnetic inclination and intensity between 1900 and 2023. Fig. S6. Contours in geomagnetic inclination and intensity between 1900 and 2023. [file 40462_2023_406_MOESM5_ESM.docx]

**Additional File 5: Changes in magnetic inclination and intensity (1900-2023)**


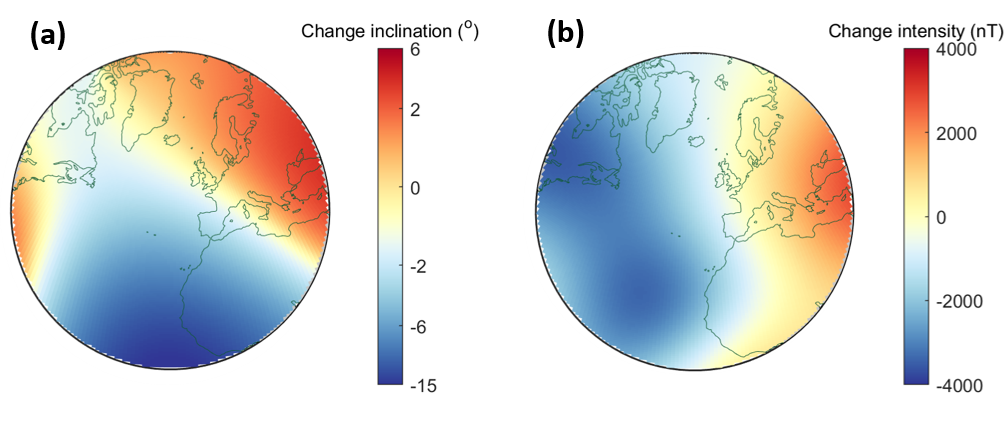


**Fig. S5:** Changes in geomagnetic inclination (**a**, degrees) and intensity (**b**, nT), between 1900 and 2023. Magnetic data are from a global IGRF modelled data of the Earth’s core-field (1,2).


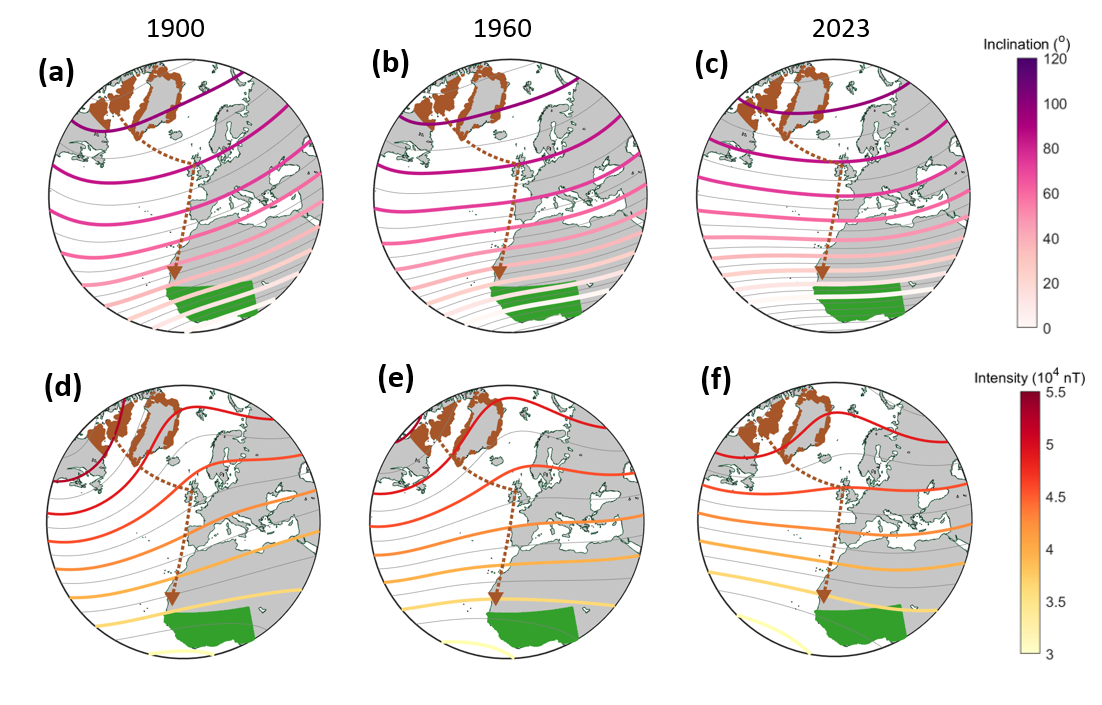


**Fig. S6:** Contour in geomagnetic inclination (**a**-**c**, degrees) and intensity (**d**-**f**, nT), in (**a**, **d**) 1900, (**b**, **e**) 1960 and (**c**, **f**) 2023. Natal (brown) and winter (green) regions of *leucorhoa* wheatears, with tracked migration (dotted brown line) as in Fig. 1. Declination changed most strongly over the period, with inclination particularly steady over W Europe. Also, inclination contours are more convex and intensity contours more concave across NW Europe. This means that a naïve *leucorhoa* migrant adapted for a *Zugknick* in the UK (as in the dotted brown line) but drifted to Norway would change its heading Southward earlier with an intensity than with an inclination signpost, reducing the risk of drifting too far East to reach the wintering grounds (migrants drifted towards the West would switch headings once on land). Magnetic data are from a global IGRF modelled data of the Earth’s core-field (1,2).

References

1. Compston D. International Geomagnetic Reference Field (IGRF) Model [Internet]. 2022. Available from: https://www.mathworks.com/matlabcentral/fileexchange/34388-international-geomagnetic-reference-field-igrf-model

2. Thébault E, Finlay CC, Beggan CD, Alken P, Aubert J, Barrois O, et al. International Geomagnetic Reference Field: the 12th generation. Earth Planet Sp. 2015 Dec;67(1):79.
